# Baicalin attenuates chronic hypoxia-induced pulmonary hypertension via adenosine A_2A_ receptor-induced SDF-1/CXCR4/PI3K/AKT signaling

**DOI:** 10.1186/s12929-017-0359-3

**Published:** 2017-08-03

**Authors:** Xiaoying Huang, Peiliang Wu, Feifei Huang, Min Xu, Mayun Chen, Kate Huang, Guo-ping Li, Manhuan Xu, Dan Yao, Liangxing Wang

**Affiliations:** 1 Division of Pulmonary Medicine, The First Affiliated Hospital of Wenzhou Medical University, Key Laboratory of Heart and Lung, Wenzhou, Zhejiang 325000 People’s Republic of China; 2 Department of Pathology, The First Affiliated Hospital of Wenzhou Medical University, Wenzhou, Zhejiang 325000 People’s Republic of China; 3 Department of Respiratory Medicine, Tongde Hospital of Zhejiang Province, Hangzhou, Zhejiang 310013 People’s Republic of China; 4 Wenzhou Medical University, Wenzhou, Zhejiang 325000 People’s Republic of China

**Keywords:** Baicalin, Pulmonary arterial hypertension, Receptor, Adenosine A_2A_, SDF-1, CXCR4

## Abstract

**Background:**

Baicalin, an important flavonoid in *Scutellaria baicalensis Georgi* extracts, exerts a variety of pharmacological effects. In this study, we explored the effects of baicalin on chronic hypoxia-induced pulmonary arterial hypertension (PAH) and investigated the mechanism underlying these effects. Moreover, we examined whether the inflammatory response was mediated by the A_2A_ receptor (A_2A_R) and stromal cell-derived factor-1 (SDF-1)/C-X-C chemokine receptor type 4 (CXCR4)-induced phosphatidyl inositol-3-kinase (PI3K) signaling in vivo.

**Methods:**

We established a hypoxia-induced pulmonary hypertension **(**HPH) mouse model by subjecting wild-type (WT) and A_2A_R knockout (A_2A_R^−/−^) animals to chronic hypoxia, and we examined the effects of a 4-week treatment with baicalin or the A_2A_R agonist CGS21680 in these animals. Invasive hemodynamic parameters, the right ventricular hypertrophy index, pulmonary congestion, the pulmonary arterial remodeling index, blood gas parameters, A_2A_R expression, and the expression of SDF-1/CXCR4/PI3K/protein kinase B (PKB; AKT) signaling components were measured.

**Results:**

Compared with WT mice, A_2A_R^−/−^ mice exhibited increased right ventricular systolic pressure (RVSP), right ventricle-to-left ventricle plus septum [RV/(LV + S)] ratio, RV weight-to-body weight (RV/BW) ratio, and lung wet weight-to-body weight (Lung/BW) ratio in the absence of an altered mean carotid arterial pressure (mCAP). These changes were accompanied by increases in pulmonary artery wall area and thickness and reductions in arterial oxygen pressure (P_a_O_2_) and hydrogen ion concentration (pH). In the HPH model, A_2A_R^−/−^ mice displayed increased CXCR4, SDF-1, phospho-PI3K, and phospho-AKT expression compared with WT mice. Treating WT and A_2A_R^−/−^ HPH mice with baicalin or CGS21680 attenuated the hypoxia-induced increases in RVSP, RV/(LV + S) and Lung/BW, as well as pulmonary arterial remodeling. Additionally, baicalin or CGS21680 alone could reverse the hypoxia-induced increases in CXCR4, SDF-1, phospho-PI3K, and phospho-AKT expression. Moreover, baicalin improved the hypoxemia induced by 4 weeks of hypoxia. Finally, we found that A_2A_R levels in WT lung tissue were enhanced by hypoxia and that baicalin up-regulated A_2A_R expression in WT hypoxic mice.

**Conclusions:**

Baicalin exerts protective effects against clinical HPH, which are partly mediated through enhanced A_2A_R activity and down-regulated SDF-1/CXCR4-induced PI3K/AKT signaling**.** Therefore, the A_2A_R may be a promising target for baicalin in treating HPH.

## Background

Pulmonary arterial hypertension (PAH) is a progressive and life-threatening disorder with a poor prognosis [1]. The disease is characterized by pulmonary vasoconstriction and increased pulmonary vascular resistance, which lead to right ventricular failure, fluid overload, and death [2]. The major histopathological features of PAH are vascular wall remodeling, in situ thrombosis, endothelial cell dysfunction and pulmonary artery smooth muscle cell (PASMC) proliferation [2, 3]. In Asian countries, PAH occurs in nearly 2 persons per 1,000,000 person-years, and PAH-related mortality occurs in 7 persons per 100 person-years [4]. However, the mechanism underlying the development of this disorder remains unknown. Increasing evidence suggests that treatments with anti-inflammatory effects, as well as treatments that can reverse cell proliferation, may be helpful for the management of PAH, but these approaches require further study.

Extracellular adenosine has anti-oxidant and anti-inflammatory properties and mediates a variety of physiological processes, including systemic vascular vasodilation and human pulmonary vessel regulation [5, 6]. The effects of adenosine are mediated by four cellular adenosine receptors: A_1_, A_2A_, A_2B_, and A_3_ [7]. Of these, the A_2A_ receptor (A_2A_R) is recognized as an important mediator of inflammatory and immune responses [8]. The A_2A_R is activated by adenosine or agonists, such as CGS21680, and initiates negative-feedback mechanisms that inhibit systemic inflammatory responses [9]. The chemokine stromal cell-derived factor-1 (SDF-1), also named CXCL12, belongs to the C-X-C chemokine subfamily. SDF-1 exerts an effective function through binding to its specific receptor, CXC chemokine receptor type 4 (CXCR4) [10]. The SDF-1/CXCR4 signaling system plays a crucial role in hematopoiesis, cardiogenesis, and vasculogenesis [11]. Specifically, the SDF-1/CXCR4 axis plays an important role in vascular remodeling. Recent evidence shows that inhibiting the SDF-1/CXCR4 axis attenuates hypoxia-induced PAH in neonatal mice by reversing pulmonary vascular cell proliferation [12]. Moreover, A_2A_R activation reportedly suppresses chemokine receptor function via heterologous desensitization, thereby enhancing the anti-inflammatory effects of adenosine [13]. Therefore, we investigated whether A_2A_R signaling affects the SDF-1/CXCR4 axis in PAH using A_2A_R knockout (A_2A_R^−/−^) mice. We hypothesized that A_2A_R activation may have a beneficial effect on PAH by down-regulating the SDF-1/CXCR4 axis. SDF-1/CXCR4 signaling triggers cell proliferation and anti-apoptosis pathways, including the phosphatidylinositol 3-kinase (PI3K)/protein kinase B (PKB; AKT) and mitogen-activated protein kinase (MAPK/ERK) pathways [14]. PI3Ks are a subfamily of lipid kinases that regulate diverse cellular processes, including proliferation, growth and survival. AKT is a central and important downstream effector of PI3K and can be activated by PI3K [15, 16]. It is worth noting that PI3K/AKT signaling plays an important role in cell migration and proliferation [17]. More importantly, PI3K/AKT activation promotes smooth muscle cell proliferation, while PI3K/AKT inhibition attenuates hypoxia-induced PAH [18]. Therefore, PI3K/AKT signaling is an important pathway downstream of SDF-1/CXCR4 that participates in regulating homeostatic cell proliferation and inflammation.

Baicalin (7-glucuronic acid, 5,6-dihydroxy-flavone) is a major flavonoid component derived from *Scutellaria baicalensis Georgi*. Numerous studies have shown that baicalin exerts broad pharmacological effects, including anti-inflammatory, anti-oxidant, anti-thrombotic, anti-tumor and anti-proliferative effects [19]. Baicalin was recently shown to not only selectively bind to chemokine ligands but also mediate anti-inflammatory effects. In addition, baicalin was found to effectively eliminate lipopolysaccharide (LPS)-induced pulmonary edema and attenuate TXA2 mimetic (U46619)-induced PAH [20]. However, the potential therapeutic effects of baicalin in PAH have not yet been investigated systematically, and the mechanisms underlying these effects have not been well elucidated.

Thus, in the present study, we examined whether baicalin exerts protective effects against PAH via the A_2A_R-induced SDF-1/CXCR4/PI3K/AKT signaling pathway in vivo. In addition, we explored the role of the A_2A_R in a hypoxia-induced PAH model using A_2A_R^−/−^ mice.

## Methods

### Reagents

Baicalin was obtained from Sigma (purity>95%; St. Louis, MO, USA). The A_2A_R agonist CGS21680 was obtained from Tocris (purity>98%; Bristol, UK). Rabbit antibodies against AKT (lot no. #4691), phospho-AKT (Ser-473, lot no. #4060), PI3K (lot no. #4257), phospho-PI3K (Tyr458, lot no. #4228), and GAPDH (lot no. #5174) were purchased from Cell Signaling Technology (Beverly, MA, USA). Mouse anti-A_2A_R (lot no. ab115250) and rabbit anti-smooth muscle myosin heavy chain 11 (MYH11) (lot no. ab53219) were purchased from Abcam (Cambridge, UK). Rabbit anti-SDF-1 (lot no. sc-28,876) and anti-CXCR4 (lot no. NB100–74396) were purchased from Santa Cruz (CA, USA) and Novus Biologicals (Littleton, CO, USA), respectively. Horseradish peroxidase (HRP)-conjugated goat anti-rabbit (lot no. A0208) and anti-mouse (lot no. A0216) IgG were obtained from Beyotime (Haimen, China). SuperSignal (R) West Femto Maximum Sensitivity Substrate and a Bicinchoninic Acid (BCA) Protein Assay Kit were purchased from Pierce (Madison, WI, USA). Tetramethyl rhodamine-5-isothiocyanate (TRITC; rhodamine)-conjugated goat anti-rabbit IgG (H&L) (lot no. ab6718) and fluorescein-5-isothiocyanate (FITC)-conjugated goat anti-rabbit IgG (H&L) (lot no. ab6717) were obtained from Abcam (Cambridge, UK). A blood gas and electrolyte fluid pack was obtained from Radiometer (Copenhagen, Denmark).

### Animals

A_2A_R-deficient (A_2A_R^−/−^) Balb/c mice were purchased from the Jackson Laboratory (Bar Harbor, ME, USA), and Balb/c mice were purchased from Slac Experimental Animal Technology (Shanghai, China). A_2A_R^−/−^ and Balb/c WT mice were bred in our specific pathogen-free animal facility, maintained under a 12-h day-night cycle, allowed free access to food and water, and housed at a temperature and humidity of 22 ± 1 °C and 60 ± 5%, respectively. For our experiments, we used 12- to 14-week-old male A_2A_R^−/−^ or Balb/c WT mice weighing 20–25 g. All experimental protocols were reviewed and approved by the Animal Ethics Committee of Wenzhou Medical University.

### Experimental protocols

The mice were divided randomly into the following seven groups (10 mice per group): WT normoxia (saline-treated) [WT(S)]; WT hypoxia (saline-treated) [WTH(S)]; WT hypoxia-plus-baicalin (baicalin, 60 mg/kg i.p.) [WTH(+Bai)]; WT hypoxia-plus-CGS21680 (CGS21680, 0.25 mg/kg i.p.) [WTH(+CGS)]; A_2A_R^−/−^ normoxia (saline-treated) [A_2A_R^−/−^(S)]; A_2A_R^−/−^ hypoxia (saline-treated) [A_2A_R^−/−^H(S)]; and A_2A_R^−/−^ hypoxia-plus-baicalin (baicalin, 60 mg/kg i.p.) [A_2A_R^−/−^H(+Bai)]. To establish a chronic hypoxia-induced PAH model, we subjected the mice to hypoxia for 28 days, as described by Huang et al. [21].In brief, the mice in the WT(S) and A_2A_R^−/−^ (S) groups were subjected to room air. The mice in the hypoxia groups were housed in a closed chamber (8 h per day), and the O_2_ concentration was dynamically maintained within a range from 9 to 11% by an automatic monitoring system.

### Invasive hemodynamic measurements

To measure the right ventricular systolic pressure (RVSP) and the mean carotid arterial pressure (mCAP), we anesthetized the mice with 20% urethane (1 ml/100 g, i.p.). A homemade polyethylene (PE) catheter (inner diameter: 0.5 mm, outer diameter: 0.9 mm) was introduced into the right jugular vein and directed to the right ventricle (RV). Another catheter was inserted into the left carotid artery. The RVSP and mCAP were recorded and analyzed using a PowerLab system (PowerLab 8/35 Multi-channel Biological Signal Recording System, AD Instruments, Colorado Springs, Australia).

### Right ventricular hypertrophy and pulmonary congestion measurements

At the end of the hemodynamic measurement period, the hearts and lungs were removed from the animals. The hearts were then divided into the RV, left ventricle (LV) and septum (S). The RV-to-(LV + S) [RV/(LV + S)] and RV-to-body weight (RV/BW) ratios were calculated as indices reflecting right ventricular hypertrophy. Additionally, the lung wet weight-to-body weight (Lung/BW) ratio was calculated to assess pulmonary congestion.

### Pulmonary arterial remodeling measurement

Lung tissue specimens obtained from the above mice were chopped into several pieces, fixed in 4% paraformaldehyde for 24 h at 4 °C, dehydrated, embedded in paraffin, and cut into 4-μm thick sections before being stained with hematoxylin-eosin (HE). The pulmonary arteries (with external diameters of 50–100 μm) were randomly selected and analyzed using Image-Pro Plus 6.0 software (Media Cybernetics, USA), and the ratios of pulmonary artery wall area to total area (WA/TA) and wall thickness to total thickness (WT/TT) were calculated to evaluate pulmonary arterial remodeling.

### Arterial blood gas analysis

On day 28 of hypoxia, the mice were anesthetized with 20% urethane. Arterial blood samples (0.5–1.0 ml) were collected from the left carotid artery using a homemade PE catheter (inner diameter: 0.5 mm, outer diameter: 0.9 mm) syringe. The blood samples were tested using an ABL90 FLEX blood gas analyzer (Radiometer, Denmark). Specifically, the hydrogen ion concentration (pH) and arterial oxygen tension (P_a_O_2_) were measured.

### Immunofluorescence detection of SDF-1 and CXCR4 protein expression in pulmonary arterioles

To assess SDF-1 and CXCR4 expression in the treated animals, we fixed the upper lobe of the right lung with 4% paraformaldehyde for 48 h, placed the lung tissues in a graded sucrose series and embedded them with Tissue-Tek OCT compound (Sakura, Japan). The 10-μm-thick sections were then fixed with ice-cold methanol for 10 min and blocked with 5% bovine serum albumin for 30 min at 37 °C before being incubated with anti-smooth muscle MYH11 antibody (1:100) overnight at 4 °C. The sections were then washed and incubated with FITC-conjugated goat anti-rabbit IgG antibody (1:500) for 1 h at room temperature before being washed 3 times with phosphate-buffered saline (PBS) and incubated with specific primary rabbit antibodies against SDF-1 and CXCR4 (1:50) overnight at 4 °C. After being washed in PBS, the sections were incubated for 1 h at room temperature with TRITC-conjugated goat anti-rabbit IgG antibody (1:500). The immunolabeled sections were observed using a Nikon Eclipse Ti microscope, and the fluorescence intensity was analyzed using Image-Pro Plus 6.0 software (Media Cybernetics, USA).

### Immunohistochemical detection of A_2A_R expression in pulmonary arterioles

The fixed lower lobes of the right lungs were removed from 4% paraformaldehyde after 24 h. To determine A_2A_R expression, we incubated the 10-μm-thick paraffin sections of lung tissue with anti-A_2A_R antibody, diluted 1:200; after an overnight incubation at 4 °C, the sections were washed three times with PBS and incubated for 1 h with HRP-conjugated goat anti-mouse IgG diluted 1:100. The sections were subsequently observed under the Nikon microscope, and the positively stained areas of the lung sections were identified using Image-Pro Plus 6.0 software (Media Cybernetics, USA).

### Western blot detection of A_2A_R, SDF-1, CXCR4, PI3K and AKT expression in lung homogenates

Frozen lung tissue specimens were homogenized with an automatic tissuelyser (MP, USA), sonicated three times, and centrifuged at 12,000 rpm for 30 min at 4 °C. The supernatant was collected at 4 °C, and the protein concentration in the supernatant was subsequently determined using a BCA Protein Assay Kit (Thermo, USA). The proteins were resolved by 10–12% SDS-PAGE. All lanes were loaded with 40 μg of protein. The proteins were then electrotransferred to PVDF membranes (Millipore, USA), which were blocked in 5% nonfat milk in PBS for 1 h at room temperature. The membranes were subsequently incubated with specific primary antibodies against A_2A_R (1:1000), SDF-1 (1:500), CXCR4 (1:1000), AKT (1:1000), phospho-AKT (1:500), PI3K (1:1000), phospho-PI3K (1:500), and GAPDH (1:1000) overnight at 4 °C before being incubated with a 1:10,000 dilution of HRP-conjugated goat anti-rabbit or anti-mouse IgG for 1 h. The membranes were visualized using a SuperSignal enhanced chemiluminescence (ECL) substrate (Pierce, USA), and then analyzed using Quantity One v-4.6.2 software (Bio-Rad Laboratories, USA).

### Statistical analysis

All values are expressed as the mean ± standard deviation (SD). Comparisons between 2 groups were analyzed by Student’s *t*-test, and multiple comparisons were analyzed by one-way analysis of variance using the least significant difference test (equal variances assumed) or Tamhane’s test (equal variances not assumed). All statistical analyses were performed using SPSS version 21.0 (IBM, Somers, NY, USA). *P* < 0.05 was considered statistically significant.

## Results

### The A_2A_R and baicalin alleviated hypoxia-induced hemodynamic changes

**Fig. 1:**
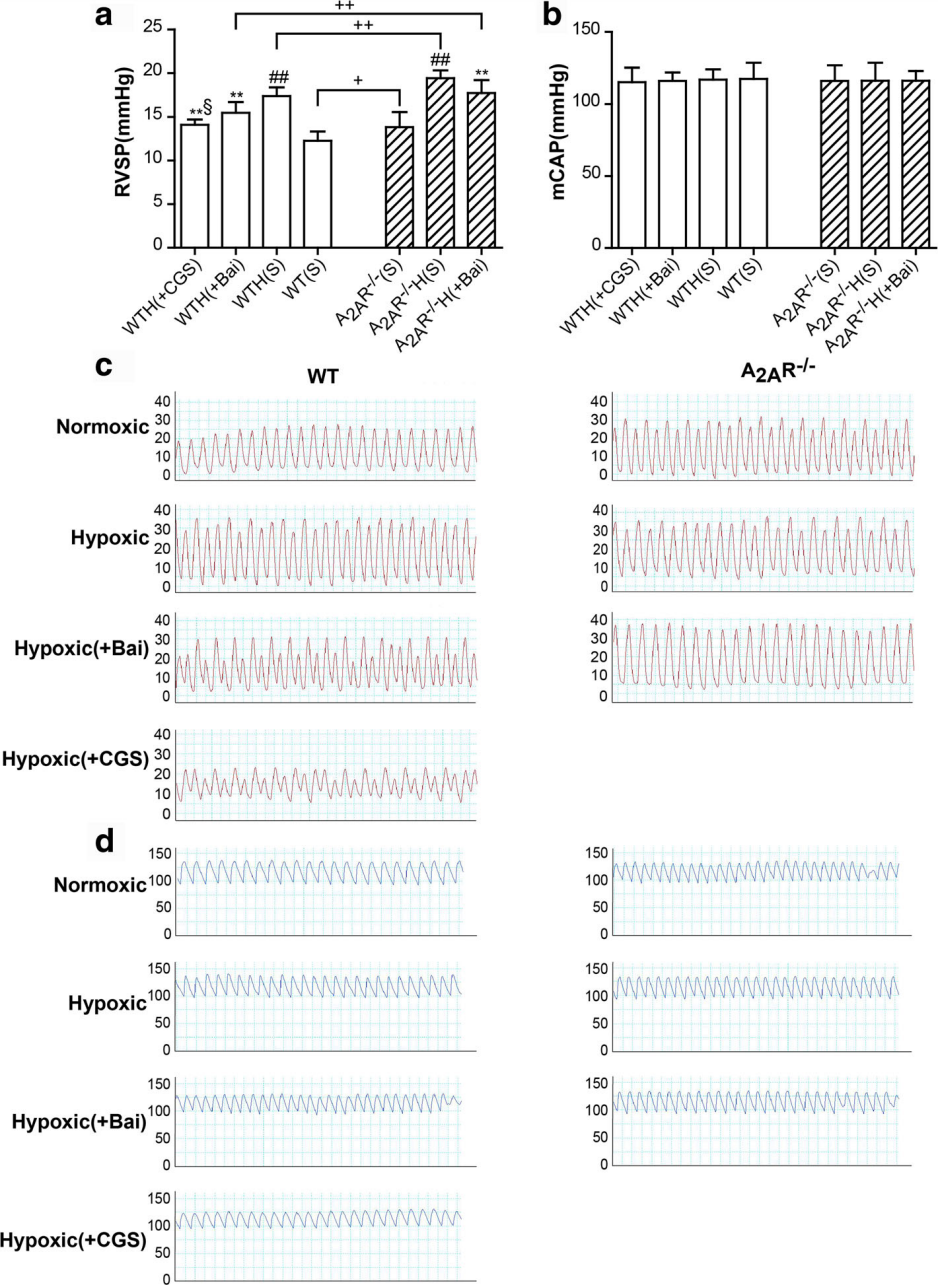
The A_2A_R and baicalin alleviated RVSP in the hypoxia-induced PAH mouse model. Effects of baicalin (+Bai, 60 mg/kg/day) and CGS21680 (+CGS, 0.25 mg/kg/day) on RVSP (**a**; *n* = 8) and mCAP (**b**; *n* = 8) in WT and A_2A_R^−/−^ mice. Representative images of RVSP waves (red) from the WT and A_2A_R^−/−^ groups (**c**). Representative images of mCAP waves (*blue*) from the WT and A_2A_R^−/−^ groups (**d**). Data are presented as the mean ± standard deviation (SD). ^#^Value significantly greater than the corresponding value in saline-treated normoxic mice (^#^ *P* < 0.05, ^##^ *P* < 0.01). *Value significantly less than the corresponding value in hypoxic mice (*P < 0.05, **P < 0.01). ^§^Value significantly less than the corresponding value in baicalin-treated mice (^§^ *P* < 0.05, ^§§^ *P* < 0.01). ^+^Value significantly greater than the corresponding value in WT saline-treated mice, WT hypoxic mice, and WT baicalin-treated mice (^+^ *P* < 0.05, ^++^ *P* < 0.01). WTH, wild-type hypoxic; A_2A_R^−/−^H, A_2A_R^−/−^ hypoxic; s, saline-treated

As shown in Fig. 1a, RVSP was much higher in hypoxic WT and A_2A_R^−/−^ mice than in nomoxic WT and A_2A_R^−/−^ mice. However, this increase in RVSP was inhibited by baicalin or CGS21680 treatment in both WT and A_2A_R^−/−^ mice. Similarly, RVSP was significantly higher in A_2A_R^−/−^(S), A_2A_R^−/−^H(S), and A_2A_R^−/−^H(Bai) mice than in WT(S), WTH(S), and WTH(Bai) mice, (*P* < 0.05, *P* < 0.01, and *P* < 0.01, respectively). However, there were no significant differences in mCAP among the 7 experimental groups (Fig. 1b).

### The A_2A_R and baicalin alleviated hypoxia-induced right ventricular hypertrophy and pulmonary congestion

**Fig. 2:**
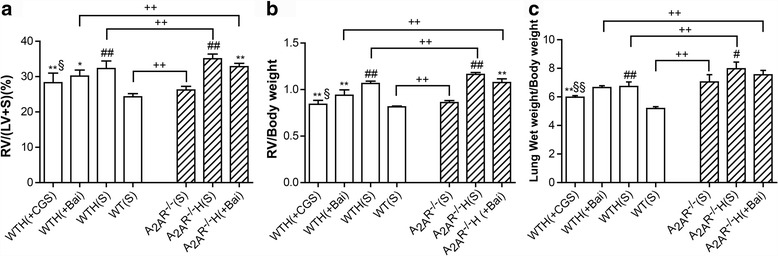
The A_2A_R and baicalin attenuated right ventricular remodeling and pulmonary congestion in the hypoxia-induced PAH mouse model. Effects of baicalin (+Bai, 60 mg/kg/day) and CGS21680 (+CGS, 0.25 mg/kg/day) on RV/(LV + S) (**a**; *n* = 8), RV/BW (**b**; *n* = 8), and Lung/BW (**c**; *n* = 8) in WT and A_2A_R^−/−^ mice. The mice were subjected to 4 weeks of hypoxia. Data are presented as the mean ± SD. ^#^Value significantly greater than the corresponding value in saline-treated normoxic mice (^#^ *P* < 0.05, ^##^ *P* < 0.01). *Value significantly less than the corresponding value in hypoxic mice (*P < 0.05, **P < 0.01). ^§^Value significantly less than the corresponding value in baicalin-treated mice (^§^ *P* < 0.05, ^§§^ *P* < 0.01). ^+^Value significantly greater than the corresponding value in WT saline-treated mice, WT hypoxic mice, and WT baicalin-treated mice (^++^ *P < 0.01*). WTH, wild-type hypoxic; A_2A_R^−/−^H, A_2A_R^−/−^ hypoxic; s, saline-treated

To investigate the effects of hypoxia on PAH in WT and A_2A_R^−/−^ mice, we measured the RV/(LV + S), RV/BW and Lung/BW ratios after 28 days of hypoxia and found that all of these parameters were increased in WT and A_2A_R^−/−^ mice. Repeated administration of baicalin (60 mg/kg/day, i.p. from day 0 to 28) significantly reduced the RV/(LV + S) and RV/BW ratios in both WT and A_2A_R^−/−^ mice without changing the Lung/BW ratio. Repeated administration of CGS21680 (0.25 mg/kg/day, i.p. from day 0 to 28) also significantly reduced the RV/(LV + S), RV/BW, and Lung/BW ratios in WT mice compared with those in WTH(S) and WTH(Bai) mice. Additionally, the RV/(LV + S), RV/BW, and Lung/BW ratios were greater in the A_2A_R^−/−^(S), A_2A_R^−/−^H(S), and A_2A_R^−/−^H(Bai) groups than in the WT(S), WTH(S), and WTH(Bai) groups (Fig. 2a–c).

### The A_2A_R and baicalin alleviated hypoxia-induced pulmonary arterial remodeling and morphological changes

**Fig. 3:**
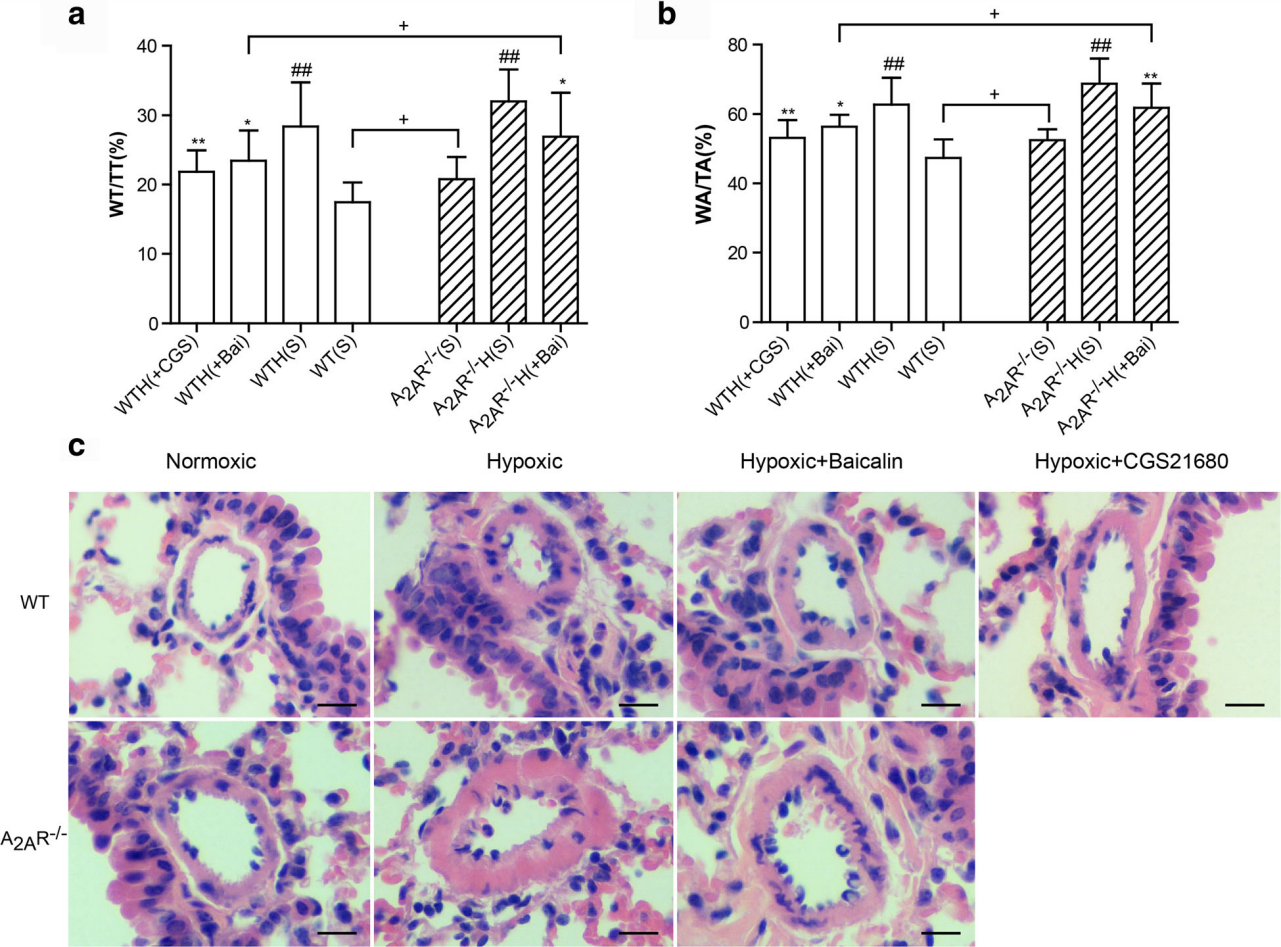
The A_2A_R and baicalin reduced pulmonary arterial remodeling in the hypoxia-induced PAH mouse model. Effects of baicalin (+Bai, 60 mg/kg/day) and CGS21680 (+CGS, 0.25 mg/kg/day) on the WT/TT(%) (**a**; *n* = 8), WA/TA(%) (**b**; *n* = 8) reversal in WT and A_2A_R^−/−^ mice. Representative images showing hypoxia-induced remodeling in the pulmonary arteries of mice in the WT and A_2A_R^−/−^ groups (**c**) (×400; scale bars indicate 50 μm), Data are presented as the mean ± SD. ^#^Value significantly greater than the corresponding value in saline-treated normoxic mice (^#^ *P* < 0.05, ^##^ *P* < 0.01). *Value significantly less than the corresponding value in mice subjected to 4 weeks of hypoxia (*P < 0.05, **P < 0.01). ^+^Value significantly greater than the corresponding value in WT saline-treated miceand WT baicalin-treated mice (^+^ *P* < 0.05). WTH, wild-type hypoxic; A_2A_R^−/−^H, A_2A_R^−/−^ hypoxic; s, saline-treated

To determine the effects of the A_2A_R and baicalin on pulmonary arterial remodeling, we investigated the pulmonary artery wall area to total area (WA/TA%) and wall thickness to total thickness (WT/TT%) by HE staining. The pulmonary artery WA/TA% and WT/TT% ratios were significantly greater in the PAH model groups compared with the control groups, i.e., the WT(S) and A_2A_R^−/−^(S) groups. While these increases were reversed by both baicalin and CGS21680 treatment, CGS21680 exerted a stronger inhibitory effect. Additionally, the pulmonary artery WA/TA% and WT/TT% ratios were significantly greater in the A_2A_R^−/−^(S) and A_2A_R^−/−^H(Bai) groups than in the WT(S) and WTH(Bai) groups. However, there were no significant differences in the pulmonary artery WA/TA% and WT/TT% ratios between the WTH(S) and A_2A_R^−/−^H(S) groups (Fig. 3a, b, and c).

### Normalizing effect of the A_2A_R and baicalin on arterial blood gas variables in the hypoxia-induced PAH mouse model

**Fig. 4:**
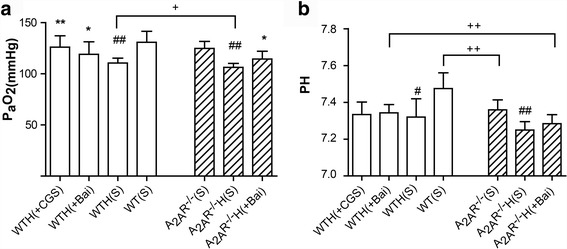
Effects of the A_2A_R and baicalin on P_a_O_2_ and pH in the hypoxia-induced PAH mouse model. Effects of baicalin (+Bai, 60 mg/kg/day) and CGS21680 (+CGS, 0.25 mg/kg/day) on P_a_O_2_ (**a**; *n* = 10) and pH (**b**; *n* = 10) in WT and A_2A_R^−/−^ mice. Data are presented as the mean ± SD. ^#^Value significantly less than the corresponding value in saline-treated normoxic mice (^#^ *P* < 0.05, ^##^ *P* < 0.01). *Value significantly greater than the corresponding value in mice subjected to 4 weeks of hypoxia (*P < 0.05, **P < 0.01). ^+^Value significantly different between WT and A_2A_R^−/−^ mice (^+^ *P* < 0.05, ^++^ *P* < 0.01). WTH, wild-type hypoxic; A_2A_R^−/−^H, A_2A_R^−/−^ hypoxic; s, saline-treated

After 4 weeks, a marked decrease in P_a_O_2_ was observed in mice subjected to chronic hypoxia compared with mice subjected to normoxia, indicating that the former mouse group had hypoxemia. Repeated administration of baicalin and CGS21680 significantly inhibited the decrease in P_a_O_2_ without affecting pH. P_a_O_2_ was significantly higher in the WTH(S) group than in the A_2A_R^−/−^ H(S) group (*P* < 0.05), and pH was significantly higher in the WT(S) and WTH(Bai) groups than in the A_2A_R^−/−^(S) and A_2A_R^−/−^H(Bai) groups (*P* < 0.01) (Fig. 4a, b).

### The A_2A_R and baicalin attenuated increases in CXCR4 expression in the hypoxia-induced PAH mouse model

**Fig. 5:**
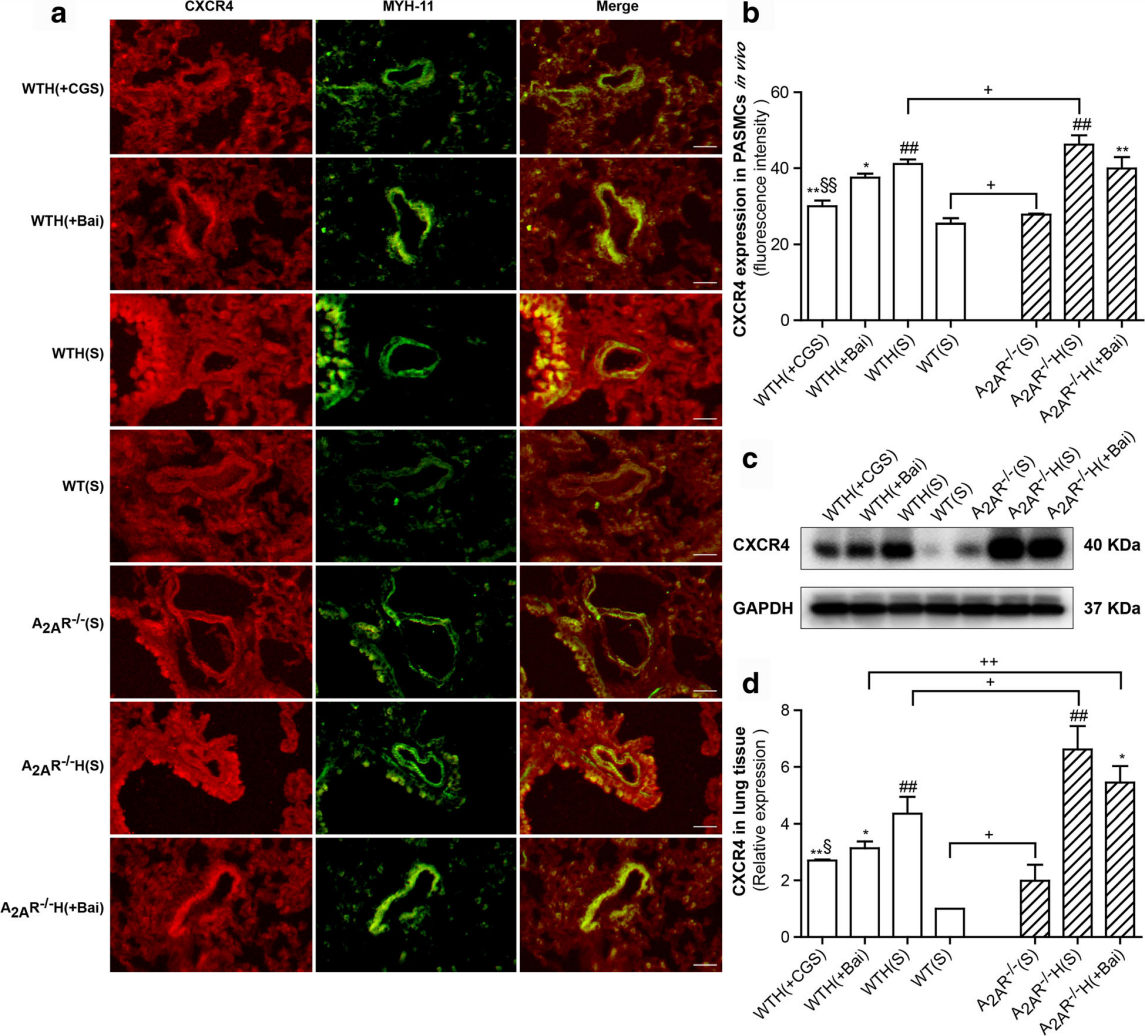
The A_2A_R and baicalin attenuated CXCR4 expression in the hypoxia-induced PAH mouse model. CXCR4 and MYH11 expression levels in mouse PASMCs were analyzed by immunofluorescence staining (**a**; *n* = 3). CXCR4 protein is stained red, and MYH11 is stained green to indicate the PASMCs (×400; scale bars indicate 50 μm). CXCR4 fluorescence intensity was calculated (**b**; *n* = 3). CXCR4 protein expression levels in lung tissue were examined by western blot. GAPDH served as an internal control (**c, d**; *n* = 3). Data are presented as the mean ± SD. ^#^Value significantly greater than the corresponding value in saline-treated normoxic mice (^##^ *P* < 0.01). *Value significantly less than the corresponding value in hypoxic mice (*P < 0.05, **P < 0.01). ^§^Value significantly less than the corresponding value in baicalin-treated mice (^§^ *P* < 0.05, ^§§^ *P* < 0.01). ^+^Value significantly different between WT and A_2A_R^−/−^ mice (^+^ *P* < 0.05, ^++^ *P* < 0.01). WTH, wild-type hypoxic; A_2A_R^−/−^H, A_2A_R^−/−^ hypoxic; s, saline-treated

To investigate the link between inflammation and the response to chronic hypoxia, we measured the effects of the A_2A_R and baicalin on CXCR4 expression in lung tissues by immunofluorescence and western blot. We performed immunofluorescence on CXCR4 and MYH11 (a marker of PASMCs). As shown in Fig. 5a and b, CXCR4 expression was significantly up-regulated in the pulmonary artery tissues of the hypoxia groups compared with the pulmonary artery tissues of the control groups. The abovementioned hypoxia-induced increases in CXCR4 expression were inhibited in the baicalin and CGS21680 treatment groups. Furthermore, the CXCR4 fluorescence intensity ratio was greater in the A_2A_R^−/−^(S), A_2A_R^−/−^H(S) groups than in the WT(S), WTH(S) mice (*P* < 0.05, *P* < 0.05, respectively); however, there was no difference in the CXCR4 ratio between the WTH(Bai) and A_2A_R^−/−^H(Bai) groups. Western blot analysis of the total lung homogenates from the WT and A_2A_R^−/−^ groups showed that hypoxia increased CXCR4 expression. Consistent with these findings, we found that treating PAH mice with baicalin or CGS21680 abolished hypoxia-induced CXCR4 expression. Compared with the A_2A_R^−/−^ groups, the WT groups displayed significantly reduced CXCR4 expression (*P* < 0.05, *P* < 0.05, and *P* < 0.01, respectively) (Fig. 5c, d).

### The A_2A_R and baicalin attenuated increases in SDF-1 expression in the hypoxia-induced PAH mouse model

**Fig. 6:**
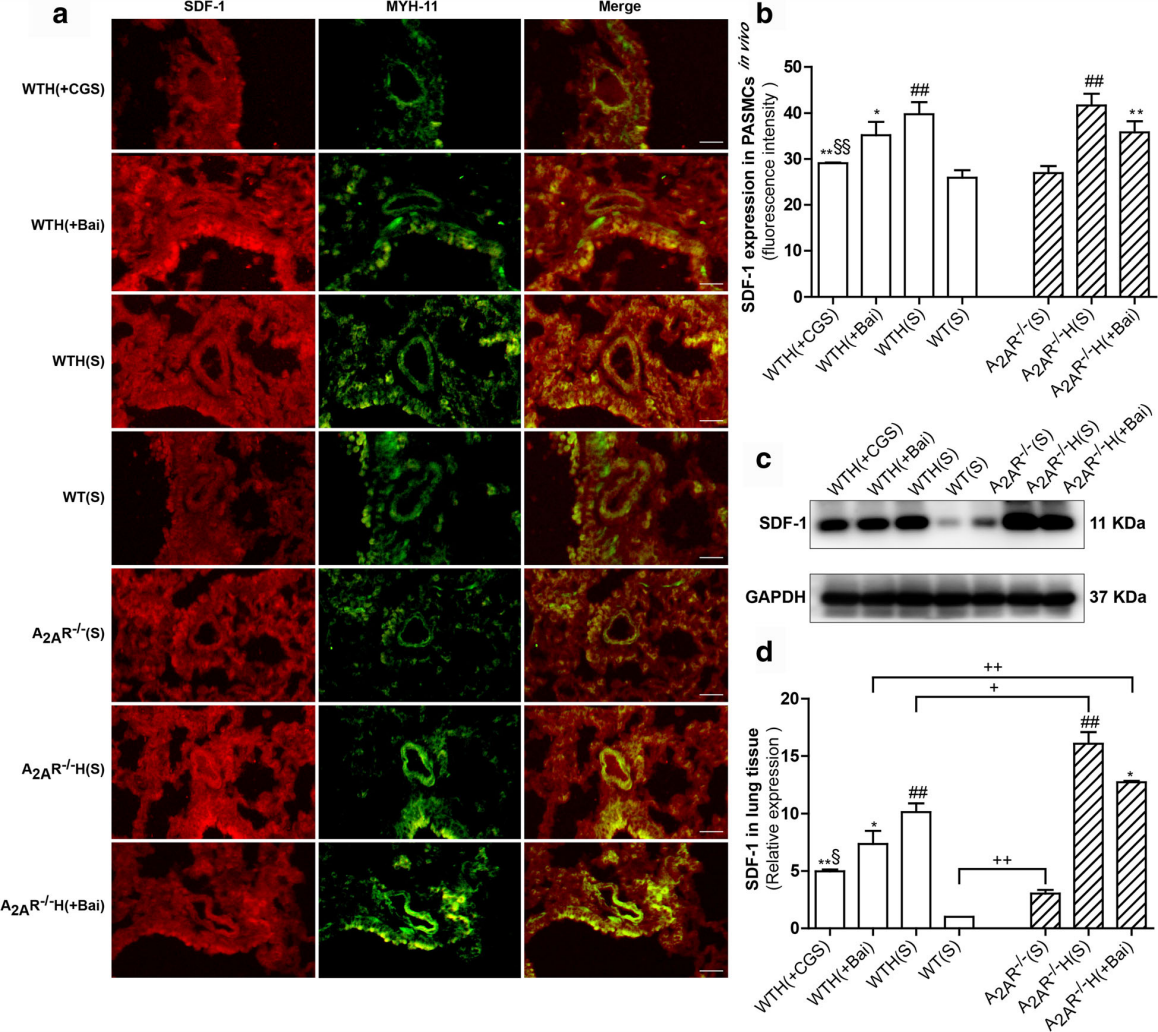
The A_2A_R and baicalin attenuated SDF-1 expression in the hypoxia-induced PAH mouse model. SDF-1 and MYH11 expression levels in mouse PASMCs were analyzed by immunofluorescence staining (**a**; *n* = 3). SDF-1 protein is stained red, and MYH11 is stained green to indicate the PASMCs (×400; scale bars indicate 50 μm). SDF-1 fluorescence intensity was calculated (**b**; *n* = 3). SDF-1 protein expression levels in lung tissues were examined by western blot. GAPDH served as an internal control (**c**, **d**; *n* = 3). Data are presented as the mean ± SD. ^#^Value significantly greater than the corresponding value in saline-treated normoxic mice (^##^ *P* < 0.01). *Value significantly less than the corresponding value in hypoxic mice (*P < 0.05, **P < 0.01). ^§^Value significantly less than the corresponding value in baicalin-treated mice (^§^ *P* < 0.05, ^§§^ *P* < 0.01). ^+^Value significantly different between WT and A_2A_R^−/−^ mice (^+^ *P* < 0.05, ^++^ *P* < 0.01). WTH, wild-type hypoxic; A_2A_R^−/−^H, A_2A_R^−/−^ hypoxic; s, saline-treated

Western blot analysis of the lung homogenates from WT and A_2A_R^−/−^ mice showed that hypoxia increased SDF-1 expression, an effect that was significantly attenuated by treatment with baicalin and CGS21680. We noted significant differences in SDF-1 expression between the WT(S), WTH(S), and WTH(Bai) groups and the A_2A_R^−/−^(S), A_2A_R^−/−^H(S), and A_2A_R^−/−^H(Bai) groups (*P* < 0.01, *P* < 0.05, and *P* < 0.01, respectively) (Fig. 6c, d). Consistent with these finding, the pulmonary artery immunofluorescence results confirmed that hypoxia increased SDF-1 expression and that baicalin and CGS21680 treatment significantly decreased hypoxia-induced SDF-1 expression. Similarly, there were no significant differences in the relative fluorescence intensity of SDF-1 between the WT(S), WTH(S), and WTH(Bai) and the A_2A_R^−/−^(S), A_2A_R^−/−^H(S), and A_2A_R^−/−^H(Bai) groups; however, compared with the WT groups, the A_2A_R^−/−^ groups exhibited slightly increased SDF-1 expression (Fig. 6a, b).

### The A_2A_R and baicalin attenuated PI3K phosphorylation and AKT phosphorylation activation in the hypoxia-induced PAH mouse model

**Fig. 7:**
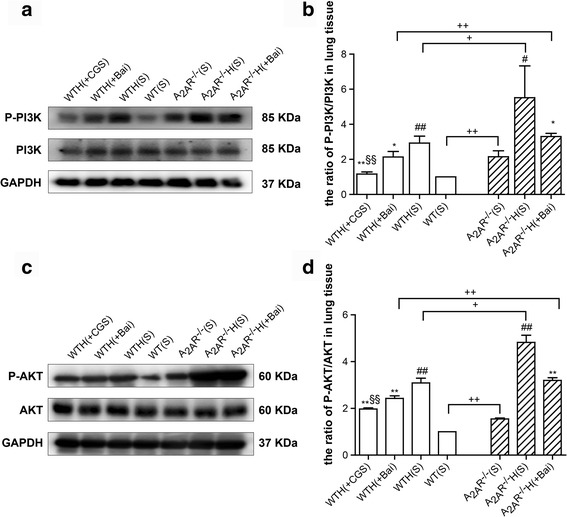
The A_2A_R and baicalin attenuated PI3K phosphorylation and AKT phosphorylation in the hypoxia-induced PAH mouse model. Phospho-PI3K levels and PI3K protein expression levels in lung tissues were examined by western blot. GAPDH served as an internal control (**a**, **b**; *n* = 3). Phospho-AKT levels and AKT protein expression levels in lung tissues were examined by western blot. GAPDH served as an internal control (**c**, **d**; *n* = 3). Data are presented as the mean ± SD. ^#^Value significantly greater than the corresponding value in saline-treated normoxic mice (^#^ *P* < 0.05, ^##^ *P* < 0.01). *Value significantly less than the corresponding value in hypoxic mice (*P < 0.05, **P < 0.01). ^§^Value significantly less than the corresponding value in baicalin-treated mice (^§§^ *P* < 0.01). ^+^Value significantly greater than the corresponding value in WT saline-treated mice, WT hypoxic mice, WT baicalin-treated mice (^+^ *P* < 0.05, ^++^ *P* < 0.01). WTH, wild-type hypoxic; A_2A_R^−/−^H, A_2A_R^−/−^ hypoxic; s, saline-treated

Our results showed that phospho-PI3K and phospho-AKT levels were higher in the WT hypoxia group than in the WT normoxia group. Baicalin significantly reduced the phospho-PI3K and phospho-AKT protein levels in hypoxic mice and exerted similar effects on the phospho-PI3K and phospho-AKT protein levels in A_2A_R^−/−^ mice. The A_2A_R agonist CGS21680 served as a positive control and decreased the phospho-PI3K and phospho-AKT protein levels in the lung tissues of the abovementioned mice to an even greater extent than did baicalin. Additionally, there were significant differences in the phospho-PI3K and phospho-AKT levels between the WT(S), WTH(S), and WTH(Bai) groups and the A_2A_R^−/−^(S), A_2A_R^−/−^H(S), and A_2A_R^−/−^H(Bai) groups (*P* < 0.01, *P* < 0.05, and *P* < 0.01, respectively). However, there were no differences in the total PI3K and AKT levels between the two sets of groups, indicating that the pathway had been activated (Fig. 7a–d).

### Baicalin increased A_2A_R expression in the hypoxia-induced PAH mouse model

**Fig. 8:**
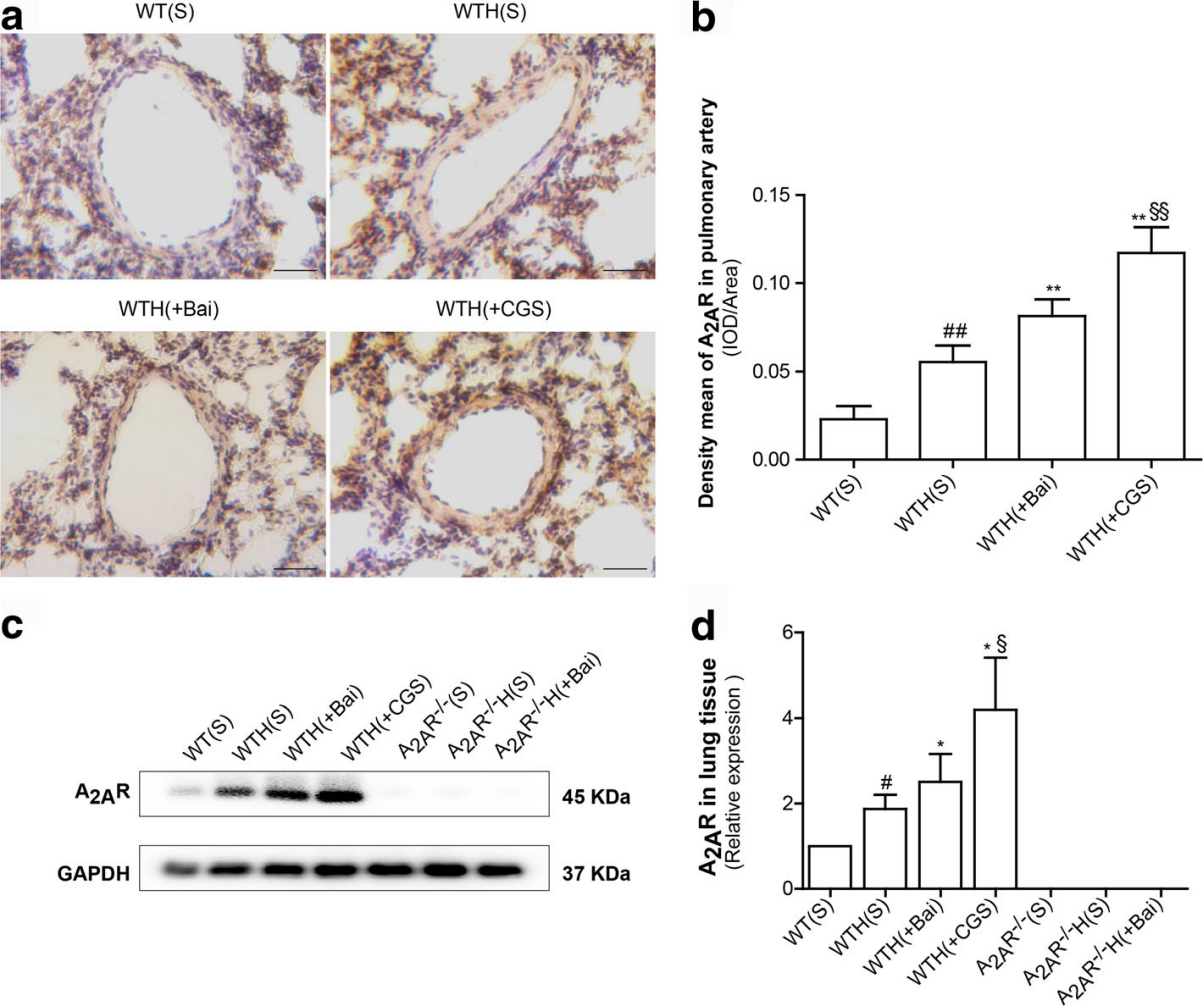
Baicalin increased A_2A_R expression in the hypoxia-induced PAH mouse model. A_2A_R protein expression levels in the pulmonary arteries were examined by immunochemical analysis with an anti-A_2A_R antibody (**a**; *n* = 10) (×400; scale bars indicate 50 μm). The mean A_2A_R density was calculated (**b**; *n* = 10). A_2A_R protein expression levels in lung tissues were examined by western blot. GAPDH served as an internal control (**c**, **d**; *n* = 3). Data are presented as the mean ± SD. ^#^Value significantly greater than the corresponding value in saline-treated normoxic mice (^#^ *P* < 0.05, ^##^ *P* < 0.01). *Value significantly greater than the corresponding value in hypoxic mice (*P < 0.05, **P < 0.01). ^§^Value significantly greater than the corresponding value in baicalin-treated mice (^§^ *P* < 0.05, ^§§^ *P* < 0.01). WTH, wildtype hypoxic; s, saline-treated

To assess the protective effects of the A_2A_R in the chronic hypoxia PAH model, we assessed the effects of baicalin on A_2A_R protein expression in lung tissue by immunohistochemical staining and western blot. A_2A_R protein expression was increased in the lungs of hypoxic WT mice, indicating that hypoxia increased A_2A_R expression in the WTH(S) group compared with that in the WT(S) group. Additionally, baicalin and CGS21680 treatment futher enhanced A_2A_R expression in the WTH(Bai) and WTH(CGS) groups, respectively (Fig. 8a–d).

## Discussion

In our study, baicalin or CGS21680 can attenuate chronic hypoxia induced increasing in RV/LV + S, RV/BW, RVSP, Lung/BW, and thickening of pulmonary arteriole in mice. Simultaneously, baicalin can significantly attenuate histopathological appearance induced by chronic hypoxia. Furthermore, the major finding of the present study is that the protective effect of baicalin was dependent on A_2A_R-related, SDF-1/CXCR4-induced PI3K/AKT signaling, as demonstrated in vivo.

PAH is a progressive and fatal disorder with a poor prognosis, and drugs that can effectively treat the disease remain lacking. HPH is characterized by continuous increases in pulmonary artery pressure, pulmonary vascular resistance and pulmonary vascular remodeling [22]. The pathology of HPH is complex. It is thought that inflammation and smooth muscle cell proliferation may play key roles in the disease. The chronic hypoxia-induced PAH mouse model has been widely used for studying pulmonary hypertension, which is characterized by increased pulmonary arterial pressure, vascular remodeling, and RV hypertrophy [21]. We successfully established a model of hypoxia-induced PAH, as demonstrated by the observed increases in RV/LV + S, RV/BW, RVSP, Lung/BW and pulmonary arteriolar thickening with luminal stenosis. Recent studies have shown that inflammation appears to play an important role in HPH. Specifically, studies have demonstrated that inflammatory cells (including macrophages, lymphocytes, neutrophils, and mast cells) and certain chemokines are closely related to the development of cell proliferation and vascular remodeling, which lead to PAH and right ventricular dysfunction [23]. Thus, inhibiting inflammation may be a useful strategy for treating PAH. SDF-1 plays an important role in regulating inflammatory cells and endothelial cell trafficking [24]. The results of many studies have indicated that the SDF-1/CXCR4 axis is closely related to pulmonary diseases, such as PAH, pulmonary fibrosis, and acute lung injury [12, 25]. In our study, we noted elevated SDF-1 and CXCR4 expression in the lungs of mice after chronic hypoxia, and these findings are consistent with those of another study [12]. In addition, the immunofluorescence results revealed SDF-1 and CXCR4 expression in PASMCs. These results demonstrated that hypoxia elicited SDF-1/CXCR4 signaling hyperactivation. Recently, Young et al. [12] reported that down-regulation of the SDF-1/CXCR4 axis significantly attenuated pulmonary hypertension in neonatal mice. However, the results of our study show that the SDF-1/CXCR4 axis plays a role in the development of HPH in WT and A_2A_R^−/−^ adult mice. It is noteworthy that there was no significant difference in pulmonary artery SDF-1 expression between the A_2A_R^−/−^(S), A_2A_R^−/−^H(S), and A_2A_R^−/−^H(Bai) groups and the WT(S), WTH(S), and WTH(Bai) groups, as demonstrated by immunofluorescence; however, SDF-1 expression in the lung homogenates was significantly higher in the A_2A_R^−/−^ groups than in the WT groups, as demonstrated by western blot. We surmised that there are two probable reasons for these findings. First, lung tissue homogenates contain not only pulmonary arteries but also pulmonary veins, alveoli, and bronchi. Second, in pulmonary hypertension, inflammatory factors act not only on pulmonary arteries but also on adjacent lung tissue. Therefore, SDF-1 levels in the lung homogenates may better reflect lung tissue inflammation than other parameters. These reasons might also account for the CXCR4 levels observed in the pulmonary artery and lung homogenates. CXCR4 is a seven-span transmembrane G-protein-coupled receptors, indicating that CXCR4 selectively binds SDF-1 to mediate intracellular signaling through the heterotrimeric G protein, which consists of three subunits: Gα, Gβ, and Gγ. In addition, the Gα subunit family is currently divided into four classes, Gαs, Gαi, Gαq, and Gα12. Meanwhile, the Gαi subunit is acting as a connecting bridge between SDF-1/CXCR4 axis and PI3K/AKT pathway [26]. Upon ligand binding to the CXCR4, the Gαi monomer triggers PI3K/AKTpathway activation [10]. SDF-1/CXCR4 signaling affects cell cycle progression and cell proliferation via the ERK and PI3K/AKT pathways [27], and PI3K/AKT signaling plays an essential role in regulating cell migration, proliferation, and survival [17]. Excessive cell proliferation is a key factor of pulmonary vascular remodeling. Notably, recent studies indicate that PI3K/AKT activation by SDF-1/CXCR4 has a major impact on cell migration and proliferation [28, 29]. Consistent with these studies, we noted that PI3K/AKT signaling was elevated in the lungs of hypoxic mice; this finding is supported by our observation of elevated phospho-PI3K and phospho-AKT levels in hypoxic mice. The abovementioned results are consistent with those pertaining to SDF-1/CXCR4 activation. However, the specific mechanism linking SDF-1/CXCR4 signaling to PI3K/AKT signaling remains unclear and requires further investigation.

Adenosine is an endogenous vasodilator, and the binding of extracellular adenosine to its receptors (A_1_R, A_2A_R, A_2B_R, and A_3_R) is known to have multiple physiological effects [7]. Saadjian et al. found that adenosine plasma levels were down-regulated in patients with PAH [30]; however, in our study, A_2A_R expression was up-regulated in the lung tissues of hypoxic mice, suggesting the presence of a negative feedback loop between adenosine and the A_2A_R may exist in PAH. Therefore, we plan elucidate the relationship between adenosine and the A_2A_R in the future. Xu et al. found that A_2A_R^−/−^ mice are more susceptible to developing PAH than other mice, indicating that this receptor exerts protective effects against PAH development [31]. In our research, A_2A_R^−/−^ mice exhibited more severe right ventricular hypertrophy and pulmonary arterial remodeling than did WT mice, suggesting that the A_2A_R plays an important role in PAH development. In addition, our understanding of the protective role played by the A_2A_R in PAH is superior to our understanding of the relationship between the A_2A_R and the SDF-1/CXCR4-PI3K/AKT pathway in PAH. In this study, we obtained the first evidence that SDF-1/CXCR4 and phospho-PI3K/phospho-AKT protein expression levels are significantly greater in A_2A_R^−/−^ mice than in WT mice, demonstrating that the A_2A_R can down-regulate the SDF-1/CXCR4-PI3K/AKT pathway in PAH.

Baicalin is a flavonoid component isolated from the flowering plant *Scutellaria baicalensis Georgi*. A recent study demonstrated that baicalin can effectively attenuate vascular smooth muscle cell proliferation by blocking the ERK1/2 signaling pathway [32]. Our previous study showed that baicalin can up-regulate ADAMTS-1 expression in chronic hypoxia to inhibit collagen Ι synthesis, thereby contributing to the attenuation of pulmonary hypertension and pulmonary vessel remodeling [33]. Baicalin can reportedly inhibit the expression of inflammatory factors in rats with acute pancreatitis [34]. However, whether baicalin can inhibit SDF-1/CXCR4 activity via the A_2A_R in HPH remains unclear. The abovementioned factors and pathways play crucial roles in the initiation of vascular remodeling. The present study showed that baicalin was effective in reducing RVSP, right ventricular hypertrophy, SDF-1/CXCR4 expression and PI3K/AKT pathway activation in PAH, thereby partially reversing pulmonary vascular remodeling. These results indicate that the interactions of baicalin with the abovementioned factors and pathways might be crucial mechanisms through which baicalin protects mice with HPH. Moreover, baicalin and CGS 21680 (an A_2A_R agonist) significantly elevated A_2A_R expression. These data indicate that baicalin exerts protective effects against HPH through the A_2A_R.

Regarding the results of our arterial blood gas analysis, we demonstrated that the PaO_2_ (hypoxemia) and pH values were significantly decreased in mice subjected to chronic hypoxia. Our results also demonstrated that baicalin and CGS21680 exert ameliorative effects on hypoxemia in mice under chronic hypoxia. To the best of our knowledge, this is the first report on the differences in arterial blood gas parameters between WT and A_2A_R^−/−^ mice and the effects of baicalin on chronically hypoxemic mice. Further studies are needed to elucidate the possible mechanisms underlying the ameliorative effects of baicalin on arterial blood gas parameters under hypoxic conditions and to clarify the differences in arterial blood gas variables between WT and A_2A_R^−/−^ mice.

## Conclusions

**Fig. 9:**
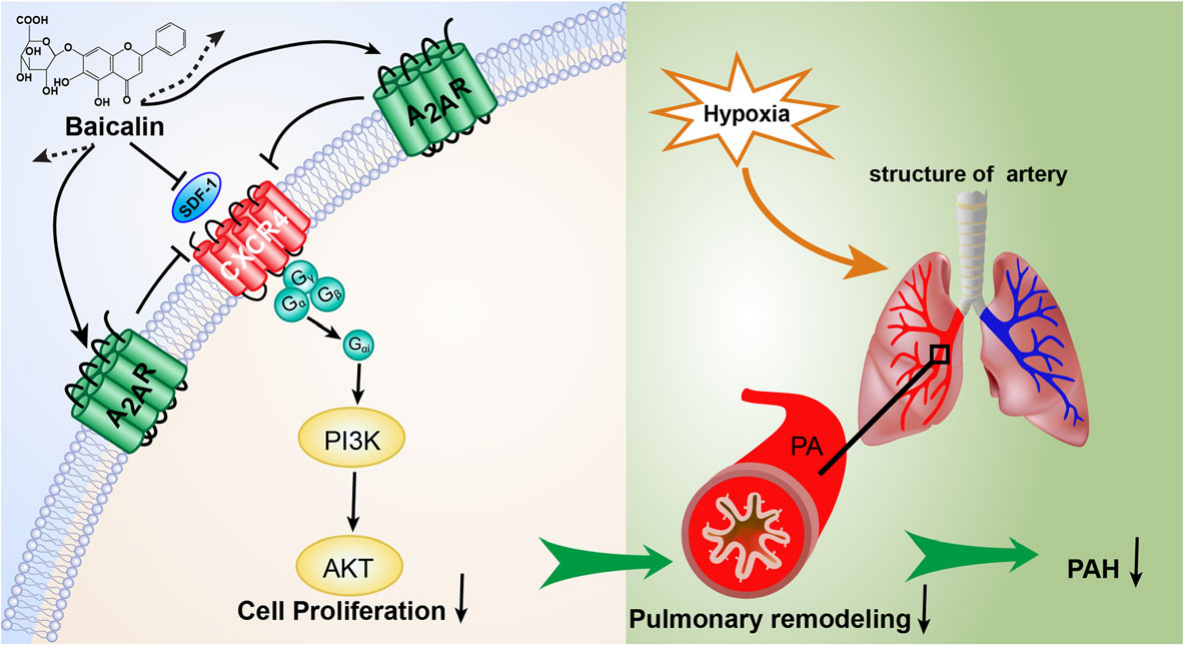
The A_2A_R and baicalin attenuated hypoxia-induced PAH. Baicalin protects against hypoxia-induced PAH in part by enhancing A_2A_R expression and then down-regulating the SDF-1/CXCR4-induced PI3K/AKT signaling pathway

Baicalin attenuates pulmonary arterial pressure, pulmonary vascular remodeling, and right ventricular hypertrophy in mice exhibiting HPH. Baicalin exerts protective effects against clinical HPH, and these effects are mediated in part through enhanced A_2A_R activity and down-regulated SDF-1/CXCR4-induced PI3K/AKT signaling (Fig. 9)**.** Therefore, the A_2A_R might be a promising target for baicalin in the treatment of HPH.

## Abbreviations

A2AR: A2A receptor
A2AR^−^/^−^: A2AR knockout
BW: body weight
CXCR4: C-X-C chemokine receptor type 4
HE: hematoxylin-eosin
HPH: hypoxia-induced pulmonary hypertension
LV: left ventricle
mCAP: mean carotid arterial pressure
MYH11: smooth muscle myosin heavy chain 11
PAH: pulmonary arterial hypertension
PaO2: arterial oxygen pressure
PASMC: pulmonary artery smooth muscle cell
pH: hydrogen ion concentration
PI3K: phosphatidyl inositol-3-kinase
RV: right ventricle
RVSP: right ventricular systolic pressure
S: septum
SDF-1: stromal cell-derived factor-1
TA: total area
TT: total thickness
WA: wall area
WT: wall thickness
WT: wild-type
